# Antioxidant, Antityrosinase, Anticholinesterase, and Nitric Oxide Inhibition Activities of Three Malaysian *Macaranga* Species

**DOI:** 10.1155/2013/312741

**Published:** 2013-11-04

**Authors:** Nor Aishah Mazlan, Ahmed Mediani, Faridah Abas, Syahida Ahmad, Khozirah Shaari, Shamsul Khamis, N. H. Lajis

**Affiliations:** ^1^Department of Food Science, Faculty of Food Science and Technology, Universiti Putra Malaysia, 43400 Serdang, Selangor, Malaysia; ^2^Laboratory of Natural Products, Institute of Bioscience, Universiti Putra Malaysia, 43400 Serdang, Selangor, Malaysia; ^3^Department of Biochemistry, Faculty of Biotechnology and Biomolecular Sciences, Universiti Putra Malaysia, 43400 Serdang, Selangor, Malaysia; ^4^Al-Moalim Bin Laden Chair for Scientific Miracles of Prophetic Medicine, Scientific Chairs Unit, Taibah University, P.O. Box 30001, Madinah al Munawarah 41311, Saudi Arabia

## Abstract

The methanol extracts of three *Macaranga* species (*M. denticulata*, *M. pruinosa,* and *M. gigantea*) were screened to evaluate their total phenolic contents and activities as cholinesterase inhibitors, nitric oxide (NO) production inhibitors, tyrosinase inhibitors, and antioxidants. The bark of *M. denticulata* showed the highest total phenolic content (2682 mg gallic acid equivalent (GAE)/100 g) and free radical scavenging activity (IC_50_ = 0.063 mg/mL). All of the samples inhibited linoleic acid peroxidation by greater than 80%, with the leaves of *M. gigantea* exhibiting the highest inhibition of 92.21%. Most of the samples exhibited significant antioxidant potential. The bark of *M. denticulata* and the leaves of both *M. pruinosa* and *M. gigantea* exhibited greater than 50% tyrosinase inhibition, with the bark of *M. denticulata* having the highest percentage of inhibition (68.7%). The bark and leaves of *M. denticulata* exhibited greater than 50% inhibition (73.82% and 54.50%, resp.) of the acetylcholinesterase enzyme (AChE), while none of the samples showed any significant inhibition of butyrylcholinesterase (BChE). Only the bark of *M. denticulata* and *M. gigantea* displayed greater than 50% inhibition of nitric oxide production in cells (81.79% and 56.51%, resp.). These bioactivities indicate that some *Macaranga* spp. have therapeutic potential in medicinal research.

## 1. Introduction

*Macaranga *is a genus from the large Euphorbiaceae family, which contains almost 300 species. This genus is commonly found in the peninsular part of forests in Malaysia and in Kalimantan, Indonesia. Approximately 27 out of the 300 species have been found in Malaysia [1]. In Malaysia, *Macaranga *is known as Mahang and has been widely used as a traditional medicine [1, 2]. For instance, a root decoction of *M. tanarius* has been used as antipyretic for fever relief and as an antitussive to suppress coughing [1]. The leaves of *M. tanarius* are used to heal wounds and relieve inflammation [3]. Similarly, a decoction of the stems and leaves of *M. denticulata *is used for washing wounds and is drunk by women after childbirth to prevent infections and cleanse the body from toxins [4]. The young shoots of *M. triloba, M. pruinosa, *and *M. gigantea* are used to treat fungal infections, while decoctions of their leaves are known to treat stomach aches [5]. In Taiwan and China, these species are incorporated into commercial products, including toothpastes and health drinks, such as herbal tea [1]. Based on these applications, *Macaranga *species are expected to possess high antioxidant activities. However, the other potential bioactivities of these species need to be investigated. 

Phytochemical and pharmacological studies on a number of *Macaranga* species have led to the isolation of flavonoids, namely, 3,7,3′,4′-tetramethylquercetin and 3,7-dimethylquercetin, which exhibit inhibition against cyclooxygenase-2 (COX-2). This inhibition of COX-2 plays an important role in chemoprevention [6]. Another flavonoid, macarangin, was isolated from *M. denticulata* and has potent antioxidant activity [4]. Recently, five ellagitannins with potential antidiabetic properties were also successfully isolated from *M. tanarius *[7]. 

Samples with high antioxidant activity can be associated with significant antityrosinase activity, as both play important roles in preventing free radical-related skin damage [2]. The antioxidant, anti-tyrosinase, and antibacterial properties of the leaves of *M. gigantea, M. pruinosa,* and *M. tanarius* have been documented [1]. However, the cholinesterase and nitric oxide inhibition activities of extracts of* Macaranga* species have not been studied, and there is little information regarding these specific properties of these extracts.

The objective of this study was to evaluate the anticholinesterase, nitric oxide generation inhibition, antioxidant and antityrosinase, activities of methanol extracts of *Macaranga *species. Fractions of varying polarity from the leaves and bark of *M. denticulata*, *M. pruinosa,* and *M. gigantea *were assessed for their medicinal and therapeutic potentials in addition to the determination of the total phenolic content of each extract.

## 2. Materials and Methods

### 2.1. Plant Materials

Leaves and bark of *M. denticulata*, *M. pruinosa,* and *M. gigantea* were collected from the Belum Forest before being deposited at the Natural Product Laboratory, Bioscience Institute, Universiti Putra Malaysia (UPM), Malaysia.

### 2.2. Chemicals and Standards

The Folin-Ciocalteau reagent (Merck, Germany), gallic acid (Sigma-Aldrich, USA), and anhydrous sodium carbonate 99% (Fluka, Switzerland) were used for TPC (total phenolic content) analysis. 1,1-Diphenyl-2-picrylhydrazyl (DPPH) and butylated hydroxytoluene (BHT) used as standards for antioxidant activity assays were purchased from Sigma-Aldrich, USA. L-3,4-Dihydroxyphenylalanine (L-DOPA), mushroom tyrosinase and kojic acid used for tyrosinase, inhibition assays were obtained from Sigma-Aldrich, USA. Acetylcholinesterase (AChE) and butyrylcholinesterase (BChE) enzymes, acetylcholine iodide, *S*-butyrylthiocholine chloride, 5,5′-dithiobis (2-nitrobenzoic acid) 99% (DTNB), and tacrine were used for both types of cholinesterase inhibition assays and were purchased from Sigma-Aldrich, USA.

### 2.3. Extraction and Fractionation of Plant Samples

Fresh leaves and bark were cut into smaller pieces, dried at room temperature, and ground into fine powders. The powdered samples (500 g) were extracted with 1 L of absolute methanol at least three times. The solvent extracts were then filtered and evaporated using a rotary evaporator. The crude extracts were then shaken with a mixture of water/methanol (2 : 1) and extracted with hexane (3 times), followed by DCM (3 times), ethyl acetate (3 times), and butanol (3 times), leaving the aqueous fraction. Each fraction was dried under reduced pressure and stored at −20°C until further use.

### 2.4. Total Phenolic Content

The total phenolic contents (TPC) of crude methanol extract samples were determined using the modified Folin-Ciocalteau method [8]. In this method, 0.5 mL of each sample (1.0 mg/mL) was introduced into a test tube followed by 0.5 mL of Folin-Ciocalteau reagent and 10 mL of 7.0% sodium carbonate. The contents of the tubes were mixed thoroughly and the reaction mixtures were allowed to stand for 1 h before measuring the absorbance of the resulting complexes at 725 nm. A standard curve was constructed for gallic acid, and the TPC values were expressed in gallic acid equivalents (GAE) in mg per 100 g. 

### 2.5. DPPH Radical Scavenging Activity

For the DPPH assays, the radical scavenging activities of the samples were determined using a modified method of Lim and Murtijaya [9]. Seven dilutions of each extract were prepared (0.01, 0.03, 0.06, 0.13, 0.25, 0.5, and 1.0 mg/mL) in triplicate in 96-well plates and 5,0 *μ*L of DPPH solution (prepared as 10 mg in 4 mL of methanol) was then added to each well. The reaction mixtures were incubated in the dark at room temperature for 30 min before reading the absorbance at 517 nm. Pure methanol was used as a blank. The antioxidant activity was expressed as the IC_50_, which is the amount of sample (in mg/mL) required to scavenge 50% of the free radicals. Thus, the extract that possesses the lowest IC_50_ value shows the highest radical scavenging capacity. BHT was used as the positive control. The percentage of inhibition was calculated as follows:

(1) $$\begin{array}{c} \%\;\text{inhibition}=\left[\frac{\text{ADPPH}-\text{ADPPH}+\text{sample}}{\text{ADPPH}}\right]\times 100. \end{array}$$

### 2.6. Ferric Thiocyanate (FTC) Assay

The FTC assay was used to evaluate the antioxidant activity as measured by the inhibition of linoleic acid peroxidation. As described by Zahin et al. [10], 4.0 mg of sample extracts in 4.0 mL of absolute ethanol were mixed with 4.1 mL of 2.5% linoleic acid in absolute ethanol, 8.0 mL of phosphate buffer (pH 7.0), and 3.9 mL of distilled water in a screw-capped amber bottle before storage at 40°C in an oven. To 1.0 mL of this solution mixture were added 9.7 mL of 75% ethanol and 0.1 mL of 30% ammonium thiocyanate. The reaction mixture was allowed to stand for exactly 3 min followed by the immediate addition of 0.1 mL of 0.02 M ferrous chloride in 3.5% hydrochloric acid (HCl) and measurement of absorbance at 500 nm. The absorbance reading was taken every 24 hours until the day after the reading of the negative control (reaction mixture without sample solution) reached a maximum. BHT quercetin and *α*-tocopherol were used as the positive controls. 

### 2.7. Antityrosinase Activity

The antityrosinase activity was determined by the modified dopachrome method as described by Lim et al. [1], using L-DOPA as the substrate. In this assay, 2 *μ*L of sample solution in DMSO (dimethyl sulfoxide) was introduced to 68 *μ*L of phosphate buffer solution (pH 6.8) in each well of a 96-well plate before adding 30 *μ*L of tyrosinase enzyme solution (0.02 mg/mL). After 5 minutes, 6.0 mM L-DOPA solution was pipetted into wells containing sample extracts. The samples (final concentration 0.5 mg/mL) were incubated at 37°C for 30 min before reading the absorbance at 490 nm. The percent inhibition was calculated as below:

(2) %  inhibition=[(Acontrol−Asample)Acontrol]×100.

Kojic acid was used as the positive control; in the reaction lacking the substrate, L-DOPA was used as the negative control.

### 2.8. Acetyl- and Butyrylcholinesterase Inhibitory Activity

For both assays, 210 *μ*L of 0.15 mM DTNB in 0.1 M phosphate buffer (pH 7.4) solution was introduced to 20 *μ*L of sample and 20 *μ*L of enzyme solution in a 96-well plate (final concentration 0.5 mg/mL). After a 10 min incubation period at room temperature, 20 *μ*L of substrate was added to each well. The absorbance was read at 412 nm at 25°C for 180 seconds. The percent inhibition was calculated as below:

(3) $$\begin{array}{c} \%\;\text{inhibition}=\left[\frac{{\text{A}}_{\text{control}}-{\text{A}}_{\text{sample}}}{{\text{A}}_{\text{control}}}\right]\times 100. \end{array}$$

For anti-AChE assays, 0.037 units/mL of AChE from electric eels and 0.25 mM of the acetylcholine iodide substrate were used. The same concentration of BChE was used for anti-BChE assays along with 0.25 mM *S*-butyrylthiocholine iodide as the substrate. Tacrine was used as the positive control.

### 2.9. Inhibition of Nitric Oxide Production

The inhibition of nitric oxide production in RAW 264.7 cells was evaluated using the Griess assay [11]. The cultured cells containing triggering agents, such as lipopolysaccharides and recombinant murine IFN-*γ*, were seeded into 96-well tissue culture plates. Then, plant extracts that had been serially diluted in DMSO (0.01, 0.03, 0.06, 0.13, 0.25, 0.5, and 1.0 mg/mL) were introduced into the wells prior to incubation for 24 hours at 37°C and 5% CO_2_ in a fully humidified incubator. After the incubation period, 50 *μ*L of supernatant from each well was transferred into 96-well plates. The Griess reagent (1% sulfanilamide and 0.1% naphthylethyenediamine dihydrochloride in 2.5% H_3_PO_4_) was added to each cell culture supernatant. The color density was measured at 550 nm using a microplate reader after a 10 minute incubation at room temperature. The percentage of inhibition was determined by comparing the inhibition of nitric oxide production from cells that had been treated with extracts with the control, which has no inhibition.

### 2.10. Statistical Analysis

Data were analyzed using MINITAB Release 14, statistical software for Windows, version 14.12.0 (Minitab Inc., USA). The results are expressed as a mean of three replicates ± SD. Differences in means were determined using ANOVA. Pearson's correlation was used to analyze the relationships between different activities.

## 3. Results and Discussion

The leaf extracts from all the three species gave extraction yields of 60–70% (w/w), while all bark extracts had lower extraction yields of 40–50% (w/w). The TPC values of the crude extracts of *Macaranga *are shown in Table 1. The bark of *M. denticulata *exhibited the highest TPC values of 2682 mg GAE/100 g, followed by the leaves of *M. pruinosa* and *M. gigantea* (2380 mg GAE/100 g and 2217 mg GAE/100 g, resp.). The bark of *M. denticulata* showed the highest antioxidant activity, with an IC_50_ value of 0.063 mg/mL, followed by the bark of *M. gigantea* and the leaves of *M. pruinosa*, with IC_50_ values of 0.145 mg/mL and 0.152 mg/mL, respectively (Table 1). These values are quite comparable to the BHT (0.052 mg/mL) and quercetin (0.019 mg/mL) standards. The hexane and DCM fractions of leaf extracts contained the highest antioxidant activities when compared to other solvent fractions. Although not observed for *M. gigantea*, the bark fractions of *M. denticulata* and *M*. *pruinosa *displayed higher antioxidant activity in the more polar solvent fractions. 

There are also various other mechanisms of antioxidant activity. The FTC assay, for example, was used to determine antioxidant activity by measuring the ability of the samples to inhibit lipid peroxidation. Most of the samples contained significant antioxidant activity in this assay displaying greater than 50% inhibition (Table 1). Additionally, greater than 90% of the active samples had 80–95% inhibition of lipid peroxidation, surpassing the standard antioxidant, *α*-tocopherol (85.11% inhibition). Only the aqueous fractions of the bark of *M. denticulata *and *M. pruinosa *exhibited antioxidant activities that were less than 50% inhibition. 

Table 2 shows that only the bark of *M. denticulata* and the leaves of *M. pruinosa* and *M. gigantea *possessed greater than 50% tyrosinase inhibition, with the bark of *M. denticulata* being the most potent inhibitor (68.7% inhibition). The leaves of both *M. pruinosa *and *M. gigantea *displayed 52.1% and 51.6% inhibition, respectively (no significant difference), while the positive control, kojic acid, had 98.51% inhibition. Only the ethyl acetate and butanol fractions of *M. denticulata* displayed greater than 50% inhibition. On the other hand, only the butanol fraction of *M. pruinosa* leaves was considered to be active, with 51.2% inhibition. The ethyl acetate fraction had 49.9% inhibition, indicating that these fractions had comparable activity. However, the leaf fractions of *M. gigantea* showed no inhibition above 50%, although the butanol fraction displayed 49.8% inhibition (Table 2). 

Only the bark and leaves of *M. denticulata *exhibited greater than 50% inhibition in the AChE inhibition assays. The bark and leaves of *M. denticulata *displayed 73.8% and 54.5% inhibition, respectively (Table 2). None of the samples exhibited more than 50% inhibition toward BChE. Tacrine, which was used as positive control for both cholinesterase assays, exhibited 98.0% inhibition toward AChE and 99.1% inhibition toward BChE. The leaf fractions of *M. denticulata *showed no significant (*P* > 0.05) inhibition of AChE. The highest inhibition value among all leaf fractions was from the hexane fraction, with 38.95%. On the other hand, only the hexane fraction of the bark of *M. denticulata* had greater than 50% inhibition, with 51.17% inhibition. This was followed by the DCM fraction, which had 47.50% inhibition. The remaining fractions displayed less than 20% inhibition (Table 2). For the Griess assay, only the bark of *M. denticulata* and *M. gigantea* showed greater than 50% inhibition at concentration 0.5 mg/mL toward NO accumulation in cells. This inhibition is not due to their cytotoxicity as indicated by their cell viability values. The bark of *M. denticulata *exhibited 81.79% inhibition, which can be considered high and has no significant (*P* > 0.05) difference from the positive control (L-DOPA), which exhibited 88.48% inhibition. This was followed by the bark of *M. gigantea*, with 56.51% inhibition, and the leaves of *M. gigantea*, with 45.15% inhibition (both have no significant difference). All of the leaf extracts were considered inactive toward NO inhibition (less than 50% inhibition). 

The reported biological activities of *Macaranga* species include antioxidant, antityrosinase, and antimicrobial activities; additionally, there is a report of a potential cancer chemopreventive agent [6, 12, 13]. The obtained results demonstrated that these three species of *Macaranga * have some therapeutic potential.

The high antioxidant activity of *M. denticulata *may be directly related to the high TPC present in its extract. The DPPH assay measures the reduction of DPPH radicals. Antioxidants scavenge the DPPH radical by donating a proton. The reduced form of DPPH exhibits a strong absorption at 517 nm [10, 14]. Polyphenols are considered to be a type of natural antioxidant. They have the ability to scavenge free radicals because of their hydroxyl group [15]. They may contribute directly toward the observed high antioxidant activity through different mechanisms exerted by different phenolic compounds or through synergistic effects with other nonphenolic compounds [14]. Hence, a high TPC value is often correlated with high antioxidant activity, though not all plant extracts exhibit the same pattern due to their different antioxidant mechanisms [10]. The bark of *M. denticulata *showed both the highest TPC value and antioxidant activity. The antioxidant level in the *M. denticulata *bark is most likely due to its high content of phenolic compounds. The FTC assay measures the level of peroxide being produced at the initial stage of linoleic acid emulsion [10, 14, 16]. The three tested species of *Macaranga* exhibited prominent antioxidant activities along with high phenolic contents; this result is in agreement with the traditional uses of these plants. The extracts also displayed high antioxidant activity after fractionation. *Macaranga* species are naturally found in secondary forests. They often recolonize the forest habitat [17]. Thus, they are often exposed to direct sunlight and may need more antioxidants to protect them, as suggested by Lim et al. [1]. Some *Macaranga* species are also myrmecophytes, meaning that they have symbiotic interactions with insects, especially ants [18]. They serve as habitats for the insects and provide food for them. In return, the insects protect their habitat from herbivores, vines, and possibly fungal infection [18]. This mutual symbiotic interaction may also lead to the potent antioxidative properties and high phenolic contents of these species. 

Tyrosinase is one of the key components in melanogenesis and the enzymatic browning of fruits, in which both reactions are undesirable. A study reported by Lim et al. [1] demonstrated that some *Macaranga* species possess high antioxidant and antityrosinase activities. Tyrosinase inhibitors are important in the cosmetic industry, especially in skin-whitening products and in treating various dermatological disorders due to the accumulation of an excessive level of epidermal pigmentation, including life-threatening melanoma [19, 20]. Presently, the search for anti-tyrosinase agents is becoming more important in both the food processing and cosmetic industries. Polyphenols have been appointed as one of the many groups of tyrosinase inhibitors [20]. Hence, plant extracts with high TPCs have a higher likelihood of yielding tyrosinase inhibitors as well as antioxidant agents. However, after fractionation, the tyrosinase inhibition effect of the extracts seemed to be diminished. For example, the extract of *M. gigantea *leaves displayed a higher percentage of inhibition (51.6%) before fractionation, as all of the fractions displayed an inhibition below 50%. It is possible that the tyrosinase inhibitor(s) act synergistically with compounds that would appear in fractions of different polarities. From the percent inhibition of the fractions from each active sample, the more polar fractions (ethyl acetate, butanol, and aqueous) seemed to exhibit a higher amount of inhibition when compared to the less polar fractions (hexane and dichloromethane). This may suggest the polarity of the active compound(s).

There are no reports of the cholinesterase inhibition properties of any *Macaranga *species. However, *Macaranga *species are expected to have AChE inhibition properties because it has been reported that plants belonging to the Euphorbiaceae family have AChE inhibitory potential [21]. There are two types of cholinesterase enzymes, AChE and BChE. Both can hydrolyze acetylcholine, although AChE has a higher preference toward this substrate. BChE, but not AChE, is also able to hydrolyze butyrylcholine [22]. Although, the role of AChE in the cholinergic system is very well recognized, the physiological function of BChE has not been systematically examined. The inhibition of AChE is suggested to be quite useful in the treatment of Alzheimer's disease and other diseases including senile dementia, ataxia, and Parkinson's disease. Alzheimer's disease is the result of a deficiency in the cholinergic system due to the rapid hydrolysis of acetylcholine. Hence, nerve impulse transmission is terminated at the cholinergic synapses. By suppressing AChE, cholinergic neurotransmission can be restored [21, 23]. Tacrine is one of the synthetic drugs used for treating the symptoms of cognitive dysfunction or memory loss associated with Alzheimer's disease. However, adverse effects have been reported for these synthetic drugs, including gastrointestinal disturbances and suppression of bioavailability [21, 23]. Both the leaves and bark of *M. denticulata *showed greater than 50% inhibition of AChE. However, after fractionation only the hexane fraction of *M. denticulata *bark extract exhibited greater than 50% inhibition. This finding suggests that the component of the extract with the activity toward AChE is present in the less polar fractions, as suggested by Orhan and Şener [23]. 

Nitric oxide (NO) signaling is one of the critical elements in normal vascular biology and many other physiological processes in addition to playing an important role in the immune system. However, the uncontrolled accumulation of nitric oxide may lead to health problems [24, 25]. NO produced by inducible nitric oxide synthase (iNOS) can contribute to many aspects of chronic inflammation. NO is described as a mediator in the inflammation process [16, 26]. Apart from inflammatory reactions, NO has also been reported to be involved in the production of melanin during UV radiation in skin cells. NO can stimulate melanocytes and increase tyrosinase activity levels [27]. Hence, the inhibition of NO may also contribute to the inhibition of tyrosinase levels. 

There is a strong linear correlation between antioxidant activity (DPPH) and TPC in *Macaranga* sp. extracts (*R* ^2^ = 0.715). Polyphenolic compounds have been associated with antioxidant activity and may directly contribute toward antioxidative action because they are effective proton donors. Phenolic compounds may also act synergistically with other nonphenolic compounds present in extracts [14]. There are also strong linear correlations between antioxidant activity and tyrosinase inhibition, AchE inhibition, and the inhibition of NO production with *R* ^2^ values of 0.787, 0.863, and 0.798, respectively. In contrast, there is a weak correlation between antioxidant activity and BChE inhibition activity (*R* ^2^ = 0.302). It can be observed that compounds with high antioxidant activities may also contribute toward the inhibition of tyrosinase, AChE, and NO production in cells. Inflammatory conditions may enhance the production of reactive oxygen/nitrogen species (ROS/NOS), which leads to oxidative stress that can damage important organic substrates. Antioxidants can scavenge free radicals and protect organisms from ROS/NOS-induced damage, leading to a reduction in inflammation [28, 29]. Antioxidants can also prevent major degenerative diseases and aging and might have protective effects toward Alzheimer's disease [30]. Oxidation-related processes coupled with tyrosinase activity can also trigger melanogenesis, which causes skin pigmentation [28, 31]. Thus, the high levels of antioxidant activity found in the plant samples may also result in a higher inhibition of tyrosinase activity, NO production, and AChE. From the obtained data, the bark of *M. denticulata* exhibited high antioxidant activity and greater than 50% inhibition of tyrosinase, AChE, and nitric oxide production. This may be due to several compounds present in the extracts acting independently or synergistically.

All of the extract samples tested displayed significant antioxidant properties even after fractionation. Thus, it can be concluded that *M. denticulata, M. pruinosa, *and *M. gigantea *have very high antioxidant activity levels. However, only *M. denticulata *(bark and leaves) has the ability to inhibit AChE, while none of the samples showed any prominent inhibition activity toward BChE. Only the bark of *M. denticulata *and *M. gigantea *showed significant inhibition of NO accumulation in cells. All three species exhibited tyrosinase inhibition properties, although their activities were weaker than that for the Kojic acid standard. Thus, the present study warrants further investigation of the active fractions of *Macaranga* sp. extracts and their active components for possible development of new classes of anti-Alzheimer and anti-inflammatory drugs.

## 4. Conclusions

Among all samples, the bark of *M. denticulata *displayed the most prominent antioxidant activity with the highest phenolic compound content. It also exhibited significant inhibition toward tyrosinase, acetylcholinesterase, and NO accumulation in cells, although it was not active toward butyrylcholinesterase. Further investigation of its active fractions should lead to the isolation and identification of the active compounds, which can contribute to the pharmaceutical and cosmetic industries.

## Figures and Tables

**Table 1:** Total phenolic content and antioxidant activity (DPPH and lipid peroxidation) of extracts and different polarity fractions of *Macaranga* species.

*Macaranga* species	Plant part	Extract/fraction	Total phenolics content (mg GAE/100 mg)^a,b^	DPPH IC_50_ value (mg/mL)^a^	Inhibition of lipid peroxidation (%)^a^
*M. denticulata *	Bark	Methanol extract	2682 ± 84.0	0.063 ± 0.008	91.03 ± 0.25
		Hexane		0.824 ± 0.016	86.01 ± 0.22
		Dichloromethane		0.316 ± 0.008	91.01 ± 1.19
		Ethylacetate		0.122 ± 0.009	91.65 ± 1.07
		Butanol		0.178 ± 0.008	91.41 ± 0.06
		Aqueous		0.285 ± 0.011	28.70 ± 0.32
	Leaves	Methanol extract	1309 ± 87.0	0.162 ± 0.011	87.94 ± 0.17
		Hexane		0.210 ± 0.007	84.87 ± 0.03
		Dichloromethane		0.246 ± 0.008	85.23 ± 2.03
		Ethylacetate		0.397 ± 0.010	90.34 ± 1.01
		Butanol		0.389 ± 0.010	91.86 ± 0.66
		Aqueous		0.378 ± 0.007	89.29 ± 0.19

*M. pruinosa *	Bark	Methanol extract	482 ± 80.0	0.203 ± 0.011	90.50 ± 0.74
		Hexane		0.510 ± 0.009	87.80 ± 1.24
		Dichloromethane		0.468 ± 0.010	88.60 ± 0.63
		Ethylacetate		0.260 ± 0.010	89.03 ± 1.07
		Butanol		0.231 ± 0.008	89.98 ± 1.04
		Aqueous		0.306 ± 0.010	22.83 ± 0.82
	Leaves	Methanol extract	2217 ± 68.0	0.152 ± 0.008	91.51 ± 0.13
		Hexane		0.163 ± 0.006	93.18 ± 0.34
		Dichloromethane		0.205 ± 0.008	91.51 ± 0.62
		Ethylacetate		0.252 ± 0.008	91.46 ± 0.71
		Butanol		0.298 ± 0.007	91.86 ± 0.42
		Aqueous		0.276 ± 0.010	92.09 ± 0.56

*M. gigantea *	Bark	Methanol extract	1220 ± 76.0	0.145 ± 0.009	91.03 ± 0.13
		Hexane		0.157 ± 0.009	86.43 ± 0.99
		Dichloromethane		0.188 ± 0.006	88.24 ± 1.12
		Ethylacetate		0.217 ± 0.009	89.16 ± 0.05
		Butanol		0.302 ± 0.010	56.01 ± 0.66
		Aqueous		0.285 ± 0.010	64.47 ± 0.57
	Leaves	Methanol extract	2380 ± 65.0	0.166 ± 0.008	92.21 ± 0.45
		Hexane		0.179 ± 0.007	90.20 ± 1.30
		Dichloromethane		0.196 ± 0.006	89.87 ± 1.01
		Ethylacetate		0.199 ± 0.008	88.50 ± 1.09
		Butanol		0.257 ± 0.011	90.12 ± 1.09
		Aqueous		0.244 ± 0.009	90.19 ± 1.08

BHT^c^			n/a	0.052 ± 0.012	91.56 ± 0.56
Quercetin^c^			n/a	0.019 ± 0.008	90.21 ± 0.37
*α*-Tocopherol^c^			n/a	0.401 ± 0.026	85.11 ± 0.57

^a^Data expressed as mean ± standard error mean (SEM) of three or more samples extracted separately.

^b^Total phenolics value was only done on crude methanol extract.

^c^Standards as positive controls.

**Table 2:** Antityrosinase, nitric oxide, acetylcholinesterase (AChE), and butyrylcholinesterase (BChE) inhibitory activity of extracts and different polarity fractions of *Macaranga* species.

*Macaranga *species	Part	Fraction	Tyrosinase inhibition (%)^a,b^	Nitric oxide inhibition (%)^a,c^	AChE inhibition (%)^a,b^	BChE inhibition (%)^a^
*M. denticulata *	Bark	Methanol extract	68.70 ± 1.48	81.79 ± 8.10	73.82 ± 2.37	24.84 ± 1.61
		Hexane	22.31 ± 1.12		51.17 ± 1.08	
		Dichloromethane	5.08 ± 1.47		47.50 ± 1.21	
		Ethy acetate	57.32 ± 1.20		15.78 ± 1.18	
		Butanol	50.99 ± 1.05		16.23 ± 1.83	
		Aqueous	48.72 ± 1.11		14.29 ± 1.38	
	Leaves	Methanol extract	27.38 ± 0.53	23.12 ± 1.88	54.50 ± 1.18	39.95 ± 1.78
		Hexane			38.95 ± 1.16	
		Dichloromethane			33.17 ± 1.98	
		Ethy acetate			5.68 ± 1.33	
		Butanol			13.99 ± 1.76	
		Aqueous			9.03 ± 1.02	

*M. pruinosa *	Bark	Methanol extract	5.87 ± 1.31	14.31 ± 2.66	23.03 ± 2.41	32.66 ± 1.8
		Hexane				
		Dichloromethane				
		Ethy acetate				
		Butanol				
		Aqueous				
	Leaves	Methanol extract	52.08 ± 0.95	15.01 ± 1.05	34.61 ± 2.53	36.58 ± 2.64
		Hexane	14.41 ± 1.38			
		Dichloromethane	23.45 ± 0.92			
		Ethy acetate	49.87 ± 1.03			
		Butanol	51.22 ± 1.54			
		Aqueous	33.45 ± 1.91			

*M. gigantea *	Bark	Methanol extract	25.43 ± 1.35	56.51 ± 2.73	33.32 ± 1.42	6.78 ± 1.35
		Hexane				
		Dichloromethane				
		Ethy acetate				
		Butanol				
		Aqueous				
	Leaves	Methanol extract	51.59 ± 1.20	45.15 ± 1.83	33.52 ± 1.09	30.53 ± 1.74
		Hexane	16.78 ± 1.31			
		Dichloromethane	30.78 ± 1.8			
		Ethy acetate	35.87 ± 0.91			
		Butanol	49.97 ± 1.07			
		Aqueous	40.32 ± 1.68			

Kojic Acid^d^			98.51 ± 1.23			
L-Name^d^				88.48 ± 1.47		
Tacrine^d^					98.01 ± 1.56	99.07 ± 1.54

^a^Data expressed as mean±standard error mean (SEM) of three or more samples extracted separately.

^b^Crude extracts that showed more than 50% inhibition were further screened for their fractions.

^c^NO inhibition assay was only done on the crude methanol extracts (0.5 mg/mL).

^d^Standards as positive controls.
